# Older Adults in the United States and COVID-19: A Qualitative Study of Perceptions, Finances, Coping, and Emotions

**DOI:** 10.3389/fpubh.2021.660536

**Published:** 2021-08-24

**Authors:** R. Turner Goins, Elizabeth Anderson, Hannah Minick, Heather Daniels

**Affiliations:** Department of Social Work, College of Health and Human Sciences, Western Carolina University, Cullowhee, NC, United States

**Keywords:** older adults, COVID-19, qualitative methods, perceptions, coping, finances, emotions

## Abstract

**Introduction:** Older adults have the poorest coronavirus (COVID-19) prognosis with the highest risk of death due to complications, making their COVID-19 experiences particularly important. Guided by the stress-appraisal-coping theoretical model, we sought to understand COVID-related perceptions and behaviors of older adults residing in the United States.

**Materials and Methods:** We used convenience sampling to recruit persons with the following inclusion criteria: Aged ≥ 65 years, English fluency, and U.S. residency. Semi structured in-depth interviews were conducted remotely and audio recorded between April 25, 2020 and May 7, 2020. Interviews were professionally transcribed with a final study sample of 43. A low-inference qualitative descriptive design was used to provide a situated understanding of participants' life experiences using their naturalistic expressions.

**Results:** The mean age of participants was 72.4 ± 6.7. Slightly over half were female (55.8%), 90.6% were White, and 18.6% lived alone. The largest percentages of participants resided in a rural area (27.9%) or small city (25.6%). We identified four themes, including (1) risk perception, (2) financial impact, (3) coping, and (4) emotions. Most participants were aware of their greater risk for poor COVID-19 outcomes but many did not believe in their increased risk. Financial circumstances because of the pandemic varied with largely no financial impacts, while others reported negative impacts and a few reported positive impacts. Coping was problem- and emotion-focused. Problem-focused coping included precautionary efforts and emotion-focused coping included creating daily structure, pursuing new and/or creative activities, connecting with others in new ways, and minimizing news media exposure. Overall, emotional health was negatively affected by the pandemic although some participants reported positive emotional experiences.

**Conclusions:** Perceiving themselves as high risk for COVID-19 complications, older adults used precautionary measures to protect themselves from contracting the virus. The precautionary measures included social isolation, which can negatively affect mental health. Older adults will need to be resourceful and draw on existing resources to cope, such as engaging in creative activities and new strategies to connect with others. Our findings underscore the importance of the preservation of mental health during extended periods of isolation by taking advantage of low-to-no-cost existing resources.

## Introduction

January 20, 2020 was the date of the first recorded case of coronavirus disease (COVID-19) in the United States (1) and 10 days later it was identified as a Public Health Emergency of International Concern by the World Health Organization (2). The U.S. President issued the Proclamation on Declaring a National Emergency Concerning the Novel Coronavirus Disease on March 13, 2020 (3). The cumulative number of confirmed and probable COVID-19 cases in the United States, as of May 20, 2021, since January 21, 2020 was 32.8 million (4). People who are aged ≥ 65 years have the poorest COVID-19 prognosis with the highest risk of death due to complications (5, 6). The highest hospitalization rates have consistently been among persons aged ≥ 65 years and the rate increases with age. As of May 19, 2021, in the U.S. there were 574,045 deaths of all ages involving COVID-19 and 458,645 or 80% were persons aged ≥ 65 years (7). Consequently, older adults were prioritized to receive a COVID-19 vaccine (8).

During the pandemic, social distancing and sheltering in place have been the main recommendations to avoid or reduce the likelihood of virus exposure (9). Further, older adults were advised to adhere to stricter social distancing directives. Centers for Disease Control and Prevention (CDC) guidance advised older adults and/or persons with underlying health conditions to limit their in-person interactions as much as possible (10). Other steps recommended by CDC for everyone included washing hands often; avoiding touching eyes, nose, or mouth; covering mouth and nose with mask when around others; and cleaning and disinfecting high frequency contact surfaces (9).

There is substantial scientific evidence with respect to the negative outcomes of social isolation. Social isolation is associated with increased loneliness, greater morbidity, and decreased quality of life as well as increased mortality risk (11). Prior to COVID-19, older adults experienced disproportionately more social isolation than younger persons (12). Mental and psychological health has been largely overlooked in response to the pandemic (13). Stress, anxiety, depressive symptoms, sleep disturbance, and loneliness are all heightened with social isolation (14, 15). Several studies have reported on mental health-related issues among older adults in the U.S. with respect to the COVID-19 pandemic (16–19). Not surprisingly, results have indicated that a large proportion of study samples report feelings of stress and loneliness (16, 18). Yet, when compared to younger adults, some research has found that older adults have experienced better mental health during the pandemic (20–22). Most of what we know so far has been epidemiological in nature with relatively less research that has qualitatively examined how older adults are responding to the pandemic (23, 24). Thus, to contextualize the published statistics, we sought to understand the responses and experiences of persons aged ≥ 65 years in the context of the COVID-19 pandemic in the United States.

## Materials and Methods

### Theoretical Model

Our study was framed within the stress-appraisal-coping theoretical model (25). Coping strategies and emotional reactions have been found to mediate the effect of the COVID-19 pandemic on stress (26). The stress-appraisal-coping theoretical model (25, 27) posits that the stress occurs when a person appraises an event as dangerous to their well-being and demands more resources than available. Cognitive appraisal, including individual characteristics, perceptions, thinking, and environmental characteristics, affect individual reactions. Coping, or a person's ongoing changing cognitive and behavioral efforts to manage stressors, also can influence stress (25). There are two types of coping in the literature: (1) Problem-solving strategies are efforts to do something active to improve a stressful situation; and (2) Emotion-focused strategies involve efforts to regulate associated emotional responses (28). Thus, we analyzed our data using this framework to better understand how cognitive appraisal and coping of older adults during COVID-19 impacts their stress response.

### Data Collection

Participants were identified and recruited using a convenience sampling approach. During spring semester 2020, 22 Masters in Social Work students taking a research course were asked to recruit and interview two persons each with the following inclusion criteria: Aged ≥ 65 years, fluent in English, and living in the U.S. Students used their personal connections to identify potential participants who they initially contacted by telephone. All the students conducted semi structured in-depth interviews with the two study participants that they identified and recruited using an interview guide (See Table 1) developed by the course professor (RTG). Given the sampling approach, most of the participants were family members of the students (e.g., parents, grandparents). All interviews were conducted remotely *via* a virtual meeting platform and audio recorded. Recordings were professionally transcribed and reviewed for accuracy. Forty-four interviews were conducted between April 25, 2020 and May 7, 2020. We excluded one interview since the participant did not meet the age criteria, yielding a total of 43 interviews analyzed for our study. The study received Western Carolina University's institutional review board approval.

**Table 1:** Interview guide.

Thank you for taking the time to speak with me about your experiences with Coronavirus or COVID-19 pandemic.
1. Can you tell me your age? and your gender?
2. Would you consider where you live… (prompts: rural? Suburban? Small city? Inner city?)
3. Have you been out of the country since mid-December 2019? *If they have, ask: What countries have you traveled since mid-December?*
4. Do you consider yourself in a “high risk category” if you contracted the Coronavirus or COVID-19?
5. Do you live alone or with others? *If they live with others, ask: Can you tell me who lives with you (not using names), relationship with you and age?*
6. Have you taken any steps or precautions to reduce your chances of contracting the Coronavirus/COVID-19?
a. *If “Yes” / If the participant has taken steps/precautions, ask:*
• When did you start taking steps/precautions to minimize your exposure to the Coronavirus/COVID-19?
• What have you done? (e.g., no longer visits with persons not in the home, quit their job, stopped volunteering, canceled appointments, stopped attending group events, bulk buying)
• How would you describe how the Coronavirus/COVID-19 pandemic has affected you in terms of your daily activities? (e.g., what are you no longer doing and what are you doing differently?)
• How would you describe how the Coronavirus/COVID-19 has affected your emotional health? *If participant reports any impact on their emotional health, ask: What are things that you are doing to help you cope during this time?*
• Have you been able to adhere to the recommended social distancing measures? *If no, ask what have they done and frequency*
• Has the pandemic negatively impacted you financially? *If so, ask:* Can you share with me how? What are you doing to manage financially during this time?
b. *If “No” / If the participant has not taken any steps/precautions:*
• Can you share with me the reasons why you haven't?
• Has the Coronavirus/COVID-19 pandemic has affected you at all in terms of daily activities? (e.g., what are you no longer doing and what are you doing differently?)
• Has the Coronavirus/COVID-19 pandemic affected your emotional health in any respects?
• Do you expect to continue to be living life as normal throughout this pandemic?
• Has the Coronavirus/COVID-19 negatively impacted you financially? *If it is has, ask: Can you share with me how?*
7. Is there anything else about your experience with the current coronavirus/COVID-19 pandemic that you would like to share that we haven't talked about already?

### Analyses

We used a low-inference qualitative descriptive design to provide a situated understanding of participants' life experiences using their naturalistic expressions (29, 30). Low-inference refers to relying on verbatim accounts of what participants said and minimizing the extent to which we as researchers reconstructed what the participants were sharing. Individual transcripts and team debriefing recordings formed the data for our analyses. A well-established mixed inductive, deductive, and reflexive analysis (31) was conducted through team processes led by a senior researcher (RTG). The analytic team consisted of four investigators with social work (LA, HM, HD), public health (RTG, HD), and gerontology (LA, RTG) perspectives. Triangulation of interpretations among this interdisciplinary team strengthened credibility of the analyses (32). Transcripts were read individually by team members using a gestalt and then line-by-line approach to *in vivo* coding using participant language to answer the question: What were the responses and experiences of COVID-19 among our participants?

The team-based analytic process consisted of individually reading each transcript, coming together to discuss words, phrases, and text segments that characterized how participants talked about their experiences. Attention was paid to what was said, the context it was offered in, and the language used. Common ideas were grouped as codes and into themes. An emergent coding schema was developed and an intra- and inter-interview theme analysis was conducted to identify emerging patterns. We used a low-inference interpretive approach to stay closer to description. Naming and meaning of themes were developed through iterative consensus discussions across the team. Investigator triangulation and an iterative design was used to ensure emergent findings were recontextualized to check meanings in subsequent interviews. An audit trail of team discussions, theme development, and the refinement of the analytic framework was maintained through audio recordings and note taking. Analyses was continued until saturation was reached, concluding that no new information would be obtained by pursuing additional interviews.

Lastly, member checking was conducted with six study participants to further enhance credibility. This involved sharing the emerging themes and interpretations with the six participants to give them an opportunity to indicate if they agreed with or if they had any feedback on the emerging themes and interpretations.

## Results

### Participant Characteristics

As shown in Table 2, the mean age of our participants was 72.4 ± 6.7, slightly more than half (55.8%) were female, 90.7% were White, and 18.6% lived alone. Persons self-identified the type of area in which they lived with the largest percentages of our study participants residing in a rural area (27.9%) or in a small city (25.6%).

**Table 2:** Participant characteristics (*n* = 43).

	***N* (%)**	**Mean ± SD**	**Range**
Age range		72.4 ± 6.7	65–92
Female	24 (55.8)		
White	39 (90.7)		
Lives alone	8 (18.6)		
**Population density**
Rural	12 (27.9)		
Small town	3 (7.0)		
Suburb	9 (20.9)		
Small city	11 (25.6)		
Large or inner city	8 (18.6)		

*SD, Standard deviation*.

#### Themes

Overall, we identified four themes with respect to responses and experiences with the COVID-19 pandemic among our participants, including (1) risk perception, (2) financial impact, (3) coping, and (4) emotions. Exemplar quotes for all themes are presented in Table 3. Brackets after quotes indicate gender (F = female, M = male) and the participant's unique identification number.

**Table 3:** Exemplar quotes.

**Risk perception**		
Yes, due to underlying conditions	They say I am. That's 69 and I've had cancer, so I guess that puts me at a higher risk. [F01]	Yes…I am a lung cancer survivor and I only have part of my lungs [F21]
Yes, because of age but with reluctance	I guess so, although I would imagine saying above 65 includes a lot of, all the way up to what, 92? I mean, up to a 100 in that case. In my own group, I think I'm a lower risk. [F02]	That's a hard question. I'm put in that category because of my age. I did have a heart attack, but apparently, my heart is healthy. My heart wasn't damaged. I've never smoked. I don't seem to have lung problems. I'm in pretty good health otherwise. So, hard to say other than that your age would be a factor so I have to be careful about that. [F12]
Yes, without reluctance but only because of age	Only because of age. [M03]	Yes, because of my age. [F04]
Yes, without elaboration	Yes. [M02]	Yes. [F23]
No, because they are healthy despite meeting age criteria	Given that they are saying anyone over 65 is a high-risk category, I would say yes. However, I believe that I'm a healthy person. And so I think that lessens the risk related to me. Because a lot of the things I've heard talk about high risk plus complicating factors, like respiratory problems, diabetes, cancer, or any of those things, which I haven't had [M08]	Not really. I'm told I am because of age, but I feel healthy for my age. [F08]
No, without elaboration	No, I don't. [F20]	I don't, but somebody else might. [M07]
**Financial impact**		
Yes, negatively	Yes, it has, because I haven't been able to work. Things get tight around because, one thing never stops, regardless of what the disease is, the bills come. [M15]	Well, yeah, I'm a real estate agent…I had 2 or 3 people that were ready or couples that were ready to buy houses right away and we were looking up until I mean like… And then one person was affiliated with a university and when they closed the university down, she just said, “I won't be looking anymore.” So, that's gone. And then others have pretty much been the same way, just wanting to wait to see how things go. And then we also have the short-term rental properties up through Airbnb and all that just got canceled immediately. So yeah, our income has definitely been affected. [F06]
Yes, positively	We're very lucky we haven't had our income affected and got a nice place to live. So mostly, I just feel lucky, and bad that other people are going through so much pain. I'm feeling guilty because we got a $2,400 check…I feel like I made money off of it. [F14]	No. If anything, it's improved things because we don't spend any money. [M14]
No impact	It hasn't affected us financially, no. [M04]	No, it hasn't. It has not. [F04]
No, not currently	No. I think we are incredibly fortunate in that we're both retired, and have an income coming in. [M08]	Day-to-day, no. Of course, you have read some retirement accounts that are smaller, but we're not terribly affected by that. [M03]
**Coping**		
**Problem-focused:**		
Reduce exposure	I rarely leave the house. Today's the first time I went shopping in a month. [M09]	Absolutely. Yes. Yeah. I mean, in fact, if I go to the grocery store and there's somebody in an aisle and even somebody who's in an aisle, I'll kind of avoid it. But, if somebody's in an aisle without a mask, I'll just walk around the next aisle over. I try to stay away from people. [F06]
Reduce susceptibility	One thing I have started doing, which I've never done in my life is to take a multivitamin every day. So, I'm doing that. [F07]	The only thing I've done is to get Intra-Cell…it's natural, homeopathic. It's good for anti-virus. Several scientific studies have been done about other viruses like HIV and so on. Recently, it was approved by China as an antiviral. [M06]
**Emotion-focused:**		
Creating daily structure	Trying to be structured. Trying to do yoga every single day. I have my own practice kind of, even though I don't go to class anymore or until this is over, but I have my own practice and that is good. [F01]	So, then I said, “Well, do you know what? I'm alive. I'm going to control my life. I'm going to be able to control, in this environment where I am now, quarantine, what I can do.” So, what I did, I started structuring my day. In the morning to about maybe 2 or 3 o'clock, I have things that I do. [F20]
New/creative activities	And there is a breathing exercise you can do to calm yourself. So, like four breaths. I can't remember that ratio, but it's four breaths in seven out and I do that because it helps me. [F09]	And I did a lot of and I still do a lot of FaceTime, and a lot of mediating, which has helped me a lot. Meditation has helped me a lot, and watching comedy. [F20]
Connecting with others in new ways	Going for walks in the neighborhood…and you can see people on the other side of the street. Everybody maintains distance. It's like a big event just to say hello to somebody other than the person that you live with. It is nice to see another human or hear another human voice and look at them when you're talking. [M05]	And then talking, we've had a couple of family and friend groups on Zoom. [M19]
Limiting news media exposure	I think you get to a point where, when I start feeling like I'm angry, I turn the TV off and I don't watch it anymore that day. [F09]	But, other than that, I stopped watching the news all day, only watch it in the morning, and then I'm done with it. [M17]
**Emotions**		
Not affected	No, I don't think so. Again, I enjoy being home. [F05]	…that's just my nature. I don't hang out with groups of people. Just in general. But the coronavirus has only made that much easier to do that. It's a haven for introverts. [M16]
Anxiety, fear, and loneliness	My anxiety level is very much elevated, little bit scared sometimes, thankful most of the time. When I get up in the morning, I take my temperature and I feel I'm OK, but very anxious about things. [F18]	I have been unable to do a lot of things I wanted to do and socialize with people. It's lonely. It's boring. I have to make up things to do. [F04]
Disappointments	Oh, pretty disconsolate about our federal response to it. [M03]	My cousins died in Jamaica, that's emotional because I can't travel to go to his funeral. He died this week as a matter of fact. [M15]
Positive feelings	I think it's been in most ways, rather positive. I don't think there's been any negatives because it's allowed me time to do more quiet activities, which I've really enjoyed, like reading and writing and things that I've always put off because I didn't have the time to do because I was always go, go, go. And now that I'm home, I can have more time for those activities and self-reflection and just quietness. [F08]	I don't feel like it has affected me a great deal. Maybe a little boredom at times, but there's just things that I would like do that I miss. But I also realized that I'm in a whole lot better shape in these things than most people are. [M18]

*F, female; M, male*.

#### Theme 1: Risk Perception

Participants were asked “Do you consider yourself in a ‘high risk category' if you contracted the Coronavirus or COVID-19?” Responses fell into six categories: (1) Yes, due to underlying health conditions; (2) Yes, because of age but with reluctance, (3) Yes, without reluctance but only because of age, (4) Yes, without elaboration, (5) No, because they are healthy despite meeting age criteria, and (6) No, without elaboration. Most of the respondents considered themselves in a high-risk category and the two most common responses were “yes, due to underlying health condition(s)” and “yes, because of age but with reluctance” in placing themselves in a high-risk category.

#### Theme 2: Financial Impact

Within the stress-appraisal-coping theoretical model, one's financial circumstances are resources that can be used and can affect how one copes. We discussed with participants the extent to which the pandemic had impacted their financial situation, and participants' discussions fell into four categories: (1) Yes, negatively; (2) Yes, positively; (3) No impact, without elaboration; and (4) No, not currently. Those who were negatively impacted had experienced a loss in their day-to-day income. Those who were positively impacted attributed it to not engaging in activities that involved spending money such as not going out to eat, shop, and/or for entertainment. There were also a few participants who shared that they benefited from the federal stimulus check.

Most our participants had not experienced a negative financial impact from the pandemic as they were retired and had a fixed income. The fourth category regarding being financially impacted were participants who reported none but also mentioned the potential of being negatively impact by losing money invested in their retirement account and the stock market. One participant discussed having temporary financial security through unemployment benefits, but was worried about possible financial insecurity once they end.

#### Theme 3: Coping

##### Problem-Focused

Participants were engaged in a variety of problem-solving strategies to avoid contracting COVID-19. These precautionary efforts were either (1)to reduce exposure to the virus or (2) to reduce susceptibility to the virus. To reduce virus exposure, all participants engaged in some of the following activities: Mask wearing, glove wearing, social distancing, handwashing, shopping at specific or designated times, and working from home. A notable number of participants described their grocery shopping experiences during the pandemic. Participants discussed avoiding people in the store, minding the 6′ distance from others, shopping at designated times for older adults, using a pre-order and pick up service, and disinfecting items upon returning home. Also, many of our study participants discussed efforts to reduce their susceptibility to the virus if exposed, including healthier eating, meditating, exercising, and taking supplements to boost their immune system.

##### Emotion-Focused

In addition to the problem-focused precautionary activities, participants enlisted emotion-focused coping strategies, that included (1) creating daily structure, (2) engaging in new or creative activities, (3) connecting with others in new ways, and (4) limiting news media exposure. Creating daily structure simply involved establishing a routine to their day. In regards to pursing new or creative activities, participants were taking care of house and/or yard projects they had put off or were starting new projects to keep them occupied. Some participants were using their time for creative pursuits such as playing an instrument or creating visual art. Several activities discussed involved food, such as cooking, baking, and/or eating. Some other activities included exercising, yoga, meditating, journaling, or deliberately spending more time outside. Participants shared how they were pursuing social engagement and support through familiar as well as new ways, including regular telephone calls, texting, and/or online video meetings. Some participants were socializing in-person with increased distance and outside, such as hosting “garden parties” or taking a walk. Lastly, to reduce their negative feelings because of the pandemic, participants shared that they were deliberately not listening to, watching, or reading the news.

#### Theme 4: Emotions

Participants discussed their emotional health in response to the pandemic. While most participants were negatively affected in some way, a few participants shared that COVID-19 had not affected their emotional health. Of those affected, anxiety, fear, and loneliness were expressed. With respect to anxiety, participants expressed overall anxiety, anxiety about the future's uncertainty, and concern about others they saw in public spaces who did not take precautionary steps such as mask wearing. There were discussions of disappointments, such as missing socializing opportunities, eating out, and visiting with loved ones. Also, with respect to disappointments, many participants were displeased with the federal government's response to the pandemic. Finally, some of our participants shared that they had experienced positive feelings, including having less stress, enjoying having more time, and a feeling a generalized sense of gratitude for what they had.

## Discussion

Most of our participants perceived themselves as in the high-risk category if they contracted COVID-19. This risk perception of the study participants makes sense as 81% of deaths due to COVID-19 are among persons aged ≥ 65 years (7). When viewed with Lazarus and Folkman's stress-appraisal-coping theoretical model, we understand that our participants cognitively appraised COVID-19 as a high-risk threat and employed significant coping skills and resources to ameliorate the emotional distress from the stress (25). There is still much to be learned about COVID-19 risk perception in older adults as study results thus far have been mixed. A study in Wuhan, China, found a higher percentage of middle-aged and older adults compared to younger adults perceived themselves as high risk for contracting COVID-19 while a slightly greater percentage of younger adults perceived themselves at high risk of death if they contracted COVID-19 (33). Prior research has found that, compared to younger adults, older adults perceived themselves at lower risk of the contracting the virus (34–36) and of dying from the virus (36).

COVID-19's financial impact has been significant, with up to 33% of people worldwide having lost income and 14% having lost a job (37). Yet, older adults have fared better financially compared to younger counterparts (38), which aligns with our findings that most of our participants were not negatively impacted financially by the pandemic. A survey of almost 5,000 U.S. adults found that across age groups, the highest percentage of those who were prepared for a financial emergency were aged ≥ 65 years. Further, this survey found that persons aged ≥ 65 years were the least likely to report losing a job and/or taking a cut in pay (38). Another U.S. study with 825 persons aged ≥ 60 years found that only 5.5% had concerns about experiencing any personal financial repercussions of the pandemic (39).

Regarding coping strategies, all our study participants engaged in both problem- and emotion-focused efforts. Problem-focused coping included precautionary steps to avoid contracting COVID-19, which corroborates other research that has shown that most older adults take the pandemic seriously. Such studies have found that older adults are the most likely to adhere to the CDC's recommendations and to engage in precautionary behaviors, including wearing a face mask, washing or sanitizing hands, keeping 6 feet distance from others, avoiding restaurants, and avoiding public or crowded places (26, 40–42).

Like other studies, our participants also coped with emotion-focused strategies, including engaging in more solitary activity (16), changing exercise regimens from group settings to home settings (43), and increasing social media use and texting (16). Moreover, our participants established low-cost coping methods such as eating healthier, taking supplements, working on projects and creative activities, finding alternatives to in-person socialization, and decreasing consumption of news media. Research examining behaviors of persons during the pandemic have found that older adults were less likely to engage in unproductive coping strategies such as substance use and behavioral disengagement compared to younger adults (26). As in other studies with older adults, and not surprisingly, our participants reported that COVID-19 has negatively affected their emotional health, including increased loneliness (16, 44), depression (45), and anxiety (20). In the general world population, the average General Anxiety Disorder score has increased (0.82–3.31) and the average Patient Health Questionnaire score has increased (0.94–2.59) (37). Yet, compared to younger persons, older adults have been found to be less likely to report depression or anxiety symptoms (20–22).

Our data provide important information about how older adults perceive the problem of COVID-19, their available resources, coping styles, and how these factors impact their emotional health. We found that while our participants perceived themselves as high risk if they contracted the virus, most of them believed they had adequate financial resources to mediate future problems related to the pandemic. This finding could explain, in part, how well our participants coped by limiting spending, minimizing COVID-19 exposure, and adopting healthier behaviors. While our participants acknowledged emotional burden, these coping skills appeared to help mitigate a more severe emotional impact the pandemic could have had on them. Lazarus and Folkman's theory may explain why others have found that older adults have not experienced as much emotional distress as younger counterparts because unlike younger counterparts, most older adults are protected by fixed incomes, Social Security, Medicare, and benefit from a lifetime of developing coping skills. It could also explain how research has found that those with financial resources are more likely to have effective skills, follow precautionary measures and recommended guidelines, and report less depression and anxiety (26, 37).

A crux of COVID-19 problem-focused coping is that the coping skill of physical distancing increases risk for isolation, a well-known risk factor among older adults for poor emotional and physical health (46, 47). While some study participants continued activities such as work, most did so from home. Participants had stopped volunteering, visiting others, eating out, or attending events and altered their grocery shopping to minimize potential virus exposure. Our findings suggest that financial stability, access to technology for socialization, access to healthy foods, and safe exercise options are important coping skills and resources to alleviate emotional distress from the stress response. Further, there are strategies that health care and social service providers can employ to help older adults address the emotional impact of COVID-19, including:

- Use a strengths perspective and praise patients who are realistic about their COVID-19 risk perception and make efforts to stay healthy and socially distance.
- Screen for loneliness, anxiety, and depression, especially among persons who live alone.
- Screen for financial impact of COVID-19. Of those who have had financial loss, recognize that is a risk factor for impaired coping and emotional health and connect to resources such as Area Agencies on Aging.
- Elicit unique coping skills before providing advice and encourage using skills that have worked for them in the past. Listen out for healthy behaviors that are being pursued such as exercise, healthier eating and supplements, and acknowledge their efforts to help build self-efficacy.
- Help identify wellness and/or exercise opportunities.
- Inquire about eating habits or conduct a nutrition screening. Refer those at risk to nutritional counseling and/or related services such as Meals on Wheels.
- If the individual does not have effective coping skills, encourage strategies such as creating daily structure, engaging in new or creative activities, connecting with others safely, and limiting news exposure.
- Recognize that it is normal for persons to experience a myriad of emotions during a pandemic, especially for those that are socially isolated. Refer those with emotional distress to effective treatments such as cognitive behavioral therapy and problem solving therapy (48).

There are several study limitations that warrant acknowledgment. These data were only collected at a single interview relatively early during the pandemic among persons residing in the U.S. Should participants have been interviewed later during the pandemic, it is likely that they would have appraised their risk differently, with changing resources such as limited capacity at hospitals and an overall slow vaccine distribution. Such circumstances may have influenced coping and emotional reactions, especially if they believed they have less control over the outcome. Also, it is possible that if more than one interview per participant was conducted, greater rapport would have been established potentially yielding more information regarding their experiences. We did not collect the state of residence of our participants. Different enacted state-level policies may have influenced the experiences and perceptions of the participants. Last, most of our participants were White, limiting our ability to examine race differences. Future research is warranted to investigate racial and ethnic differences with COVID-19 experiences, including Blacks, American Indians, Alaska Natives, and Latinx. Research has found that these groups have been found to be more likely to contract the virus and to experience greater negative health effects that the general U.S. population (49–52).

These insights into risk perceptions, financial resources, coping strategies, and emotional health have public health implications. Studies prior to the COVID-19 pandemic indicate that older adults were at increased risk for social isolation and loneliness, which can lead to physical and emotional problems (46). Clearly, the pandemic has presented greater challenges for older adults as well as for their health care and social service providers. The COVID-19 pandemic has raised concerns with respect to reduced physical activity, limited use of services, increased anxiety, and compromised nutrition among older adults (15). We heard that our participants were being resourceful in their coping although concerted efforts are needed to bolster programs and services that support older adults. Further, such programs and services are now tasked with developing new and creative ways to reach their patients and/or clients. Such efforts, for instance, can help with high speed internet access, provide support regarding technology to connect to their social network, increasing use of telemedicine and telepsychiatry, providing home delivered meals, and distributing the COVID-19 vaccine.

## Data Availability Statement

The raw data supporting the conclusions of this article will be made available by the authors, without undue reservation.

## Ethics Statement

The studies involving human participants were reviewed and approved by Western Carolina University Institutional Review Board. Written informed consent for participation was not required for this study in accordance with the national legislation and the institutional requirements.

## Author Contributions

RG contributed to the conception, study design, and interview guide. HM and HD conducted interviews and member checking. RG, HM, and HD conducted the qualitative analysis. RG, HM, HD, and EA wrote sections of the manuscript. All authors contributed to the manuscript revision, read, and approved the submitted version.

## Conflict of Interest

The authors declare that the research was conducted in the absence of any commercial or financial relationships that could be construed as a potential conflict of interest.

## Publisher's Note

All claims expressed in this article are solely those of the authors and do not necessarily represent those of their affiliated organizations, or those of the publisher, the editors and the reviewers. Any product that may be evaluated in this article, or claim that may be made by its manufacturer, is not guaranteed or endorsed by the publisher.
